# Hemophagocytic Lymphohistiocytosis After Inactivated Influenza Vaccination in a Young Man Complicated by Severe Rhabdomyolysis

**DOI:** 10.7759/cureus.23334

**Published:** 2022-03-20

**Authors:** Sara Soliman, Anastasia Bakulina

**Affiliations:** 1 Internal Medicine, Yale School of Medicine, Waterbury, USA

**Keywords:** rhabdomyolysis, anakinra, high ferritin, influenza vaccine, hemophagocytic lymphohistiocytosis (hlh)

## Abstract

Hemophagocytic lymphohistiocytosis (HLH) is an immune response disorder that is usually fatal despite treatment. It is characterized by a dysregulation in natural killer (NK) T-cell function, causing activation of lymphocytes and histiocytes, resulting in a cytokine storm, end-organ damage, and eventually death. In this report, we describe the case of a previously healthy 38-year-old Caucasian man who presented with fever, nausea, vomiting, abdominal pain, myalgias, and weight loss for one week after inactivated influenza vaccination. The initial evaluation showed leukocytosis, lactic acidosis, and a severely elevated creatine kinase level (19,639 IU/L). The presentation was consistent with a diagnosis of sepsis, likely secondary to viral etiology and rhabdomyolysis. Subsequently, he rapidly deteriorated, requiring mechanical ventilation and developed refractory shock requiring pressor support and continuous veno-venous hemofiltration for acute kidney injury due to rhabdomyolysis. Later, he developed bicytopenia, hyperferritinemia, hypertriglyceridemia, and elevated inflammatory markers, raising the possibility of underlying HLH. Further tests showed low NK cell cytotoxicity and elevated sCD25. The H-score, which is a clinical tool to estimate the probability of HLH, showed an 88-93% probability of that potentially fatal disorder.

The patient was treated with pulse-dose corticosteroids, intravenous immunoglobulins (IVIGs), and anakinra. He had a prolonged and complicated hospital stay for about two months. However, he was able to slowly recover. We believe that he developed secondary HLH in the setting of vaccination. Although rare, an early suspicion of HLH leads to the early initiation of directed therapy with immunosuppressant that would limit morbidity and mortality.

## Introduction

Hemophagocytic lymphohistiocytosis (HLH) is a severe inflammatory immune state induced by activated macrophages and cytotoxic cells, causing a highly fatal syndrome [1]. It causes immune dysregulation, which results in impaired function of cytotoxic T lymphocytes (CTLs) and natural killer (NK) cells, leading to excess activated macrophages and, consequently, cytokine storm and multi-organ dysfunction. HLH was historically divided into “primary or familial” and “secondary or acquired”. This classification aimed to distinguish highly fatal cases of HLH during infancy from milder cases that were present later in life. Primary HLH refers to patients with a family history or genetic mutations, while secondary HLH refers to conditions triggered by an insult such as infections or malignancy. However, HLH can be present in later life also. Infections can trigger both primary and secondary HLH, and adult cases can be highly fatal as well [1,2]. The clinical features of HLH are similar to a number of common infectious and noninfectious conditions that cause fever, pancytopenia, hepatic abnormalities, or neurologic findings, thus, diagnosis is usually delayed. The H-score can help predict the possibility of HLH and direct early initiation of appropriate treatment.

## Case presentation

We present the case of a previously healthy 38-year-old Caucasian man who was evaluated for intense epigastric pain, nausea, vomiting, and diarrhea one week after he received an inactivated influenza virus vaccination. He also developed myalgia, oral aphthous ulcers, and a weight loss of 5 kg during that period. He denied recent sick contacts or travel outside of New England and has not started any new medications. He identified himself as men who have sex with men (MSM). His last sexual contact was four months ago, and recent HIV testing was negative. His family history was notable for several family members who had systemic lupus erythematosus and inflammatory arthritis.

Initial assessment revealed a normal body temperature, heart rate of 140/minute, respiratory rate of 20/minute, blood pressure of 170/130 mm Hg, and oxygen saturation of 100% on room air. Physical exam showed erythema of eyelids, oral aphthous ulcers, oral petechiae on a background of pale mucosa, nonpruritic petechial rash over his anterior chest, and mild-to-moderate epigastric tenderness. The rest of the physical examination was unremarkable. Initial work-up at the time of the presentation is shown in Table 1 ("day 1" column).

**Table 1:** Laboratory work-up including complete blood count and complete metabolic panel. Figure 1 shows laboratory work-up on presentation, day 5 and day 10. Note leukocytosis, thrombocytopenia, and transaminitis rapidly rising CK levels. BUN: blood urea nitrogen, CRP: C-reactive protein, CK: creatine kinase, ALT: alanine transaminase, AST: aspartate transaminase, LDH: lactate dehydrogenase.

Complete blood count			
	Day 1	Day 5	Day 10
WBC	13 thous/mm^3^	24 thous/mm^3^	14 thous/mm^4^
RBC	6.74 mill/mm^3^	4.45 mill/mm^3^	2.6 mill/mm^4^
Hemoglobin	19.4 g/dL	12.7 g/dL	7.6 g/dL
Hematocrit	56.5%	37%	3%
Platelets	157 thous/mm^3^	77 thous/mm^3^	59 thous/mm^4^
Blood chemistry			
	Day 1	Day 5	Day 10
Sodium	131 mmol/L	127 mmol/L	135 mmol/L
Potassium	5.1 mmol/L	6.9 mmol/L	4.1 mmol/L
Chloride	93 mmol/L	95 mmol/L	98 mmol/L
CO_2_	31 mmol/L	21 mmol/L	23 mmol/L
Anion gap	7 mmol/L	11 mmol/L	13 mmol/L
BUN	25 mg/dL	62 mg/dL	60 mg/dL
Creatinine	0.65 mg/dL	3.6 mg/dL	2.4 mg/dL
Lactic acid	2.5 mmol/L	2.5 mmol/L	1.8 mmol/L
Ferritin	430 ng/mL	2,550 ng/mL	1,055 ng/mL
CRP	14 mg/L		59 mg/L
CK	19,639 IU/L	>160,000 IU/L	81,418 IU/L
Total bilirubin	1.2 mg/dL	1.5 mg/dL	0.9 mg/dL
ALT	294 IU/L	294 IU/L	735 IU/L
AST	883 IU/L	883 IU/L	1,841 IU/L
Alkaline phosphatase	84 IU/L	84 IU/L	109 IU/L
LDH	2,593 IU/L		
Lipase	44 IU/L		

The patient was started on IV fluids with improvements of his vital signs and clearance of lactic acid. Lab work showed markedly elevated CK levels and myoglobinuria on microscopic urine analysis, consistent with rhabdomyolysis.

Although he was initially afebrile, he eventually spiked a fever up to 104.6 F. His presentation with systemic illness manifested with GI symptoms, oral ulcers, generalized aches, and rhabdomyolysis was suggestive of possible viral infection complicated by rhabdomyolysis.

During the course of his hospitalization, he has rapidly deteriorated. On hospital day 3, he was found unresponsive. Arterial blood analysis showed severe metabolic acidosis (PH 7.20, HCO_3_ 11 mmol/L). He was intubated and transferred to the ICU. Soon after the transfer, he developed shock, requiring multiple vasopressor agents despite ongoing aggressive IV fluid resuscitation.

**Figure 1:**
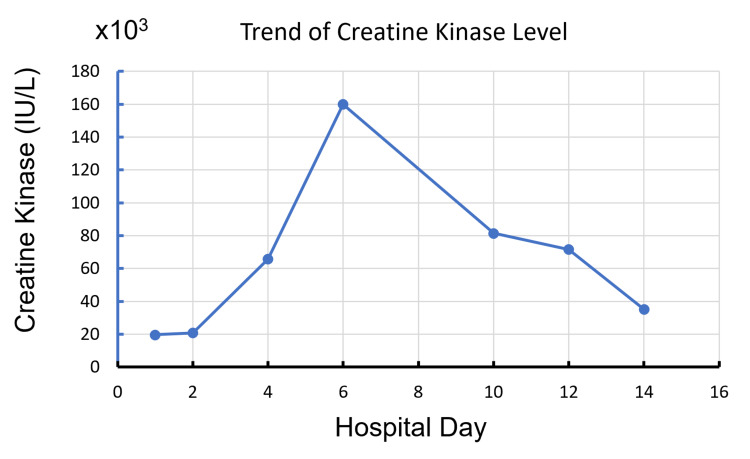
Trend of creatine kinase levels during hospitalization.

The subsequent laboratory work-up showed progressive elevation of CK levels (the trend is shown in Figure 1), acute renal failure, and hyperkalemia. Unfortunately, ongoing muscle damage and kidney failure caused persistent hyperkalemia despite hemodialysis. The patient required continuous veno-venous hemofiltration. Laboratory work-up during that time is shown in Table 1 ("day 5" column).

The underlying cause of the patient’s presentation was unclear. He had an extensive infectious work-up, included in Table 2. Repeated blood cultures were negative. A viral blood panel was only positive for Epstein-Barr virus (EBV) DNA. EBV IgG was positive but IgM was negative, consistent with past infection. A respiratory viral panel by nasopharyngeal swab showed positive rhinovirus. A CT of the abdomen and pelvis didn’t reveal any pathology. Due to ongoing severe acute respiratory distress syndrome (ARDS), the patient's serum was also tested for β-d-glucan, which appeared to be positive. Subsequently, aspergillus antigen was obtained and was negative. Further work-up of β-d-glucan warranted bronchoscopy, which was negative for *Pneumocystis jiroveci* infection.

**Table 2:** Infectious work-up. The table shows extensive infectious work-up showing positive EBV and CMV IgG. The respiratory viral panel was only positive for rhinovirus. RPR: rapid plasma reagin, PCR: polymerase chain reaction, EBV: Epstein-Barr virus, CMV: cytomegalovirus, HSV: Herpes simplex virus, RSV: respiratory syncytial virus.

Multiple blood cultures	Negative
Rapid strep test (throat)	Negative
RPR qual.	Negative
Lyme disease IgM, IgG	Negative
Urine Legionella antigen	Negative
*Neisseria gonorrhoeae* RNA (swab)	Negative
*Chlamydia trachomatis* RNA (swab)	Negative
Babesia, Anaplasma, Ehrlichia smears	Negative
Anaplasma, Ehrlichia PCR	Negative
HIV RNA	Negative
HIV viral load	No detected
EBV IgM, IgG	Negative, positive (>750 U/mL)
CMV IgM, IgG	Negative, positive
HSV 1 IgM, HSV 2 IgM	Negative
HSV 1/HSV 2 PCR	Negative
Coxsackie serology	Negative
COVID-19 PCR	Negative
Influenza A&B Ag	Negative
Influenza A&B PCR	Negative
Adenovirus PCR	Negative
RSV PCR	Negative
Metapneumovirus PCR	Negative
Rhinovirus PCR	Positive
Coronaviruses 229E, NL63	Negative
Parainfluenza PCR	Negative
Viral hepatitis panel	Negative
West Nile virus IgM	Negative

Given that his clinical picture could also be triggered by an immune disorder, he underwent additional testing as autoimmune serologies including antinuclear antibody (ANA), rheumatoid factor, and antineutrophil cytoplasmic antibodies (ANCA), which were negative. Complement levels were normal as well. (Results are shown in Table 3.) Given severe muscle injury, the patient was also worked up for possible autoimmune myopathies. Anti-PL-7, anti-PL-12, and anti-Mi-2 were all negative (Table 4).

**Table 3:** Rheumatological work-up. Extensive rheumatological work-up was unrevealing. ANA: antinuclear antibody, SRP: signal recognition particle, ESR: erythrocyte sedimentation rate, ANCA: antineutrophil cytoplasmic antibodies.

ESR	3 mm/hr
Rheumatoid factor	<8.6 IU/mL
ANA	Negative
C3 complement	108 mg/dL (88-165)
C4 complement	29 mg/dL (14-44)
Anti-SRP	Negative
ESR	3 mm/hr
ANCA screen	Negative
Autoantibodies to proteinase-3	Not detected
Autoantibodies to myeloperoxidase	Not detected

**Table 4:** Myositis panel. Work-up for autoimmune myositis is negative.

Myositis panel	
Anti-Jo-1	<0.1 Negative
Anti-PL 7	Not detected
Anti-PL 12	Not detected
Anti-MI 2	Not detected
Anti-KU	Not detected
Anti-EJ	Not detected
Anti-OJ	Not detected

**Figure 2:**
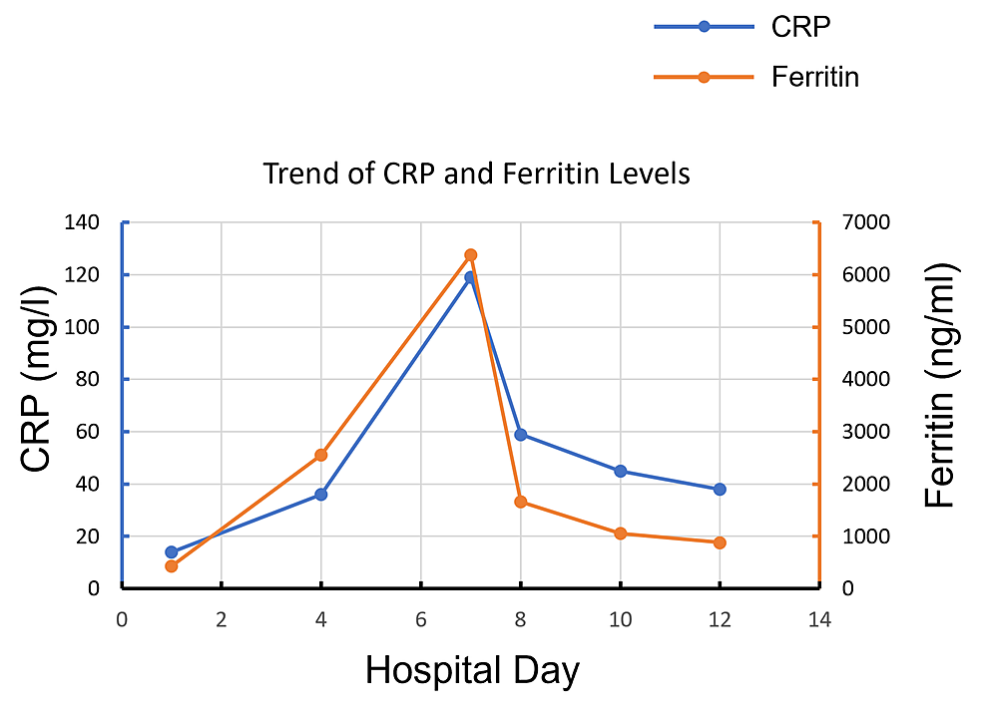
Trends of CRP and ferritin levels during hospitalization. CRP: C-reactive protein.

Although ferritin level was initially normal, it has significantly risen, as well as C-reactive protein (Figure 2). Notably, his inflammatory markers spiked on hospital days 6-7 as well as CK level. Fasting lipid profile showed hypertriglyceridemia (triglycerides level peaked at 568 mg/dl).

Marked hyperferritinemia in the context of severe systemic inflammatory response raised the suspicion of possible secondary HLH, especially given the recent immunogenic trigger of influenza vaccine and/or possible active viral infection. In addition to elevated C-reactive protein and ferritin, the immunologic panel revealed elevated soluble interleukin-2 receptors (sCD25) and depressed NK cell function (Table 5), which is pathognomonic for HLH. HLH work-up is included in Table 6.

**Table 5:** Natural killers function assay. NK: natural killer.

Natural killer function assay		
	Result	Reference range
NK 50:1	1	≥20%
NK 25:1	2	≥10%
NK 12:1	2	≥5%
NK 6:1	1	≥1%
NK lytic units	0	≥2.6 units
CD16/CD56% (NK cells)	2	%
	Interpretation: decreased to absent NK function.	

**Table 6:** HLH diagnostic criteria. Our patient has fulfilled the 6/8 criteria. HLH: hemophagocytic lymphohistiocytosis, PLT: platelets.

The diagnosis of HLH can be established if criterion 1 or 2 is fulfilled
A molecular diagnosis consistent with HLH or five out of eight diagnostic criteria are fulfilled
Fever
Splenomegaly
Cytopenias (two or more cell lines: Hgb <9.0 g/dL, PLT <100×10^9^/L, neutrophils <100×10^9^/L)
Hypertriglyceridemia and/or hypofibrinogenemia
Hemophagocytosis in bone marrow/spleen/lymph nodes without evidence of malignancy
Low or no NK cell activity
Ferritin >500 pg/L
sCD25 >2400 U/mL
Our patient has fulfilled six out of the eight criteria

HLH was confirmed based on HLH-2004 diagnostic criteria, fulfilling six out of the eight criteria and the HLH-probability calculator. For further work-up for HLH, after extended infectious and immunological work-up, flow cytometry revealed absolute CD19^+^ B-cell lymphopenia as well as absolute CD3^+^ T-cell lymphopenia, which are nonspecific findings. However, there was no evidence of monoclonal B-cell lymphoproliferative disease. Given that a genetic component might have contributed to the patient’s clinical therapy, extended gene testing was done with no evidence of HLH triggering genes. It was thought that HLH had probably developed secondary to an inactivated influenza vaccine. The patient was started on directed therapy according to the HLH protocol. He received pulse dose methylprednisolone 1000 mg for three days followed by a slow steroid taper. Given hypogammaglobulinemia, he was started on intravenous immunoglobulin (IVIG) 10 g weekly to maintain IgG above 700 mg/dL. IgG levels were monitored weekly with slow improvement back to baseline. Unfortunately, he developed severe complications with prolonged steroid use, including life-threatening GI bleed from peptic ulcer and steroids were quickly tapered, he was started on anakinra, a steroid-sparing agent resulting in the slow gradual improvement of clinical status and inflammatory markers trend. After two months, he was discharged to a rehabilitation center where he continued to receive IVIG monthly and daily anakinra.

## Discussion

Hemophagocytic lymphohistiocytosis (HLH) is a life-threatening condition characterized by a sustained and progressive immune response. The most common trigger for secondary HLH is infection. Other important causes include malignancies and rheumatological diseases in which the condition is termed macrophage activation syndrome (MAS). Viral infections are mostly implicated, with EBV viremia being the most common virus reported in HLH cases [1,2]. The condition is sufficiently common, with an incidence of about 1/50,000. The pathophysiology of HLH is complex. It involves the excessive activation of antigen-presenting cells (histiocytes and macrophages) as well as T-cells, causing persistently elevated circulating levels of multiple pro-inflammatory cytokines, including interferon (IFN-γ), interleukin (IL-6), IL-10, IL-12, IL-16, IL-18, and tumor necrosis factor (TNF-α) resulting in progressive organ dysfunction and potentially death. NK cell dysfunction is a characteristic pathologic finding in HLH [2,3]. NK cells maintain an appropriate immunological response to noxious stimuli by modulating the response of antigen-presenting cells. This helps the human body control autoimmune diseases as well as severe reactions to infectious insults as viruses. Abnormal NK cell responses and natural killer T (NKT) cell deficiency result in a state of persistently activated macrophages, causing cytokine storm and organ dysfunction [3].

Features of HLH are nonspecific, resulting in a delayed or missed diagnosis. The main symptoms include fever, jaundice, hepatitis, splenomegaly, lymphadenopathy, rash, and neurological signs. Diagnosis of hemophagocytic syndrome relies on clinical, laboratory, and histopathological findings. Cytopenia and markedly elevated ferritin levels are the hallmark laboratory findings in HLH. Other abnormal laboratory findings include severe liver dysfunction, which manifests as hypertriglyceridemia, hypofibrinogenemia and elevated serum transaminases, hyponatremia and hypoproteinemia are also seen. Two highly specific parameters are the increased alpha chain of the interleukin-2 receptor (sCD25) and impaired NK-cell activity. Histopathological evaluation of the bone marrow or other affected organs (spleen, lymph nodes) can show significant histiocytosis with evidence of hemophagocytic activity for which the condition was named [1,2,4].

The diagnosis of HLH is based upon the H-score, the first validated score proposed by the Histiocyte Society in 1991 to predict the possibility of HLH. The score comprised eight clinical, biologic, and cytologic variables. The diagnosis requires meeting five of eight criteria for diagnosis. Most of the criteria are nonspecific, especially fever and splenomegaly [1,5]. However, markedly elevated ferritin levels (>10,000 microgram/L) seem to be fairly specific for diagnosis [6]. Our patient had most of the above-mentioned features, including fever, hepatitis, and high ferritin. However, he did not have any evidence of organomegaly on serial abdominal CT scans. The calculated H-score was 206 points, which suggested an 88-93% probability of HLH.

Evidence of hemophagocytosis is one of the criteria that support the diagnosis of HLH. Bone marrow biopsies can also help rule out other diseases with similar clinical presentations. However, hemophagocytosis is not specific to HLH and can be seen in other conditions such as sepsis and after blood transfusion. A study of 64 marrow core biopsy specimens and aspirates from 58 patients with clinical suspicion for secondary HLH showed a poor correlation between histological evidence of hemophagocytosis and disease probability [7]. In our patient, a bone marrow biopsy was deferred given high clinical suspicion of HLH. Since the condition is highly fatal, it is important to initiate therapy even if not all the criteria are met.

Our patient had an extensive work-up to exclude underlying causes of HLH. Infectious work-up was only evident for positive rhinovirus on the respiratory viral panel, immunologic work-up was unrevealing, and there was no evidence of lymphoproliferative disorders on flow cytometry. Finally, gene testing was inconclusive as well. Given the temporal relationship between vaccine administration and the patient’s symptoms, we believe the influenza vaccine was the trigger of HLH in this case. Although very rare, few cases have reported vaccine-induced HLH. After a review of the literature, we found only one other case report that described a case of HLH that happened following influenza vaccination in a patient with aplastic anemia [8]. Other cases have also reported HLH following COVID vaccination [9].

The development of rhabdomyolysis, in this case, is quite interesting. Influenza vaccine-related rhabdomyolysis has been previously described in the literature [10]. Similar to our case, these cases describe patients with rhabdomyolysis onset within one week after vaccination. However, most of the cases we found were associated with concurrent use of lipid-lowering drugs, either statins or fibrates [10,11]. In these cases, CK elevation varied from mild to severe. Some authors accounted for adjuvants in inactivated influenza vaccines as possible triggers of rhabdomyolysis [12].

We found only one case report that describes the coincidence of HLH and rhabdomyolysis in a young woman with typhoid fever who has recovered from her severe illness with antibiotics and supportive treatment [13]. In our case, due to the lack of academic data, it is unclear if these conditions developed independently from each other or if they are linked pathophysiologically.

Treatment aims to induce remission by inhibiting the release of cytokines. According to the HLH-2004 protocol, those patients are aggressively treated with dexamethasone, etoposide, and cyclosporine A. All agents have strong inhibitory effects on cytotoxic T cells and macrophages. The protocol was designed mainly for children, and treatment in adults requires a more individualized approach [14]. Steroids are the main core of treatment and should be started in all patients. Our patient received a high dose of intravenous methylprednisolone 1 gm for three days with a plan for slow dexamethasone taper according to the HLH-2004 protocol (initially 10 mg/m^2^ for two weeks followed by 5 mg/m^2^ for two weeks, 2.5 mg/m^2^ for two weeks, 1.25 mg/m^2^ for one week followed by one week of tapering). However, he developed severe side effects from high-dose steroids, including multiple peptic ulcers leading to life-threatening GI bleeding; thus, steroids were quickly tapered and anakinra was added. Anakinra is a recombinant interleukin (IL) 1 receptor antagonist with a strong anti-inflammatory effect, which can be used in combination with steroids or IVIG in the case where patients show minimal improvement or as a steroid-sparing agent. A study of eight critically ill patients showed anakinra in combination with IVIG and/or corticosteroids resulted in a hospital survival rate of 50% without significant toxicity [15].

Intravenous polyvalent immunoglobulins (IVIGs) in HLH work as a support for defective humoral immunity as well as part of the targeted anti-inflammatory treatment regimen. High therapeutic doses (1.6 g/kg over two and three days) inhibit the immune response through inhibition of complement activation and neutralization of cytokines [14]. Given the evidence of hypogammaglobulinemia, he was started on immunoglobulins weekly in combination with daily anakinra. Despite his prolonged and eventful hospitalization, he was discharged to a rehabilitation center after two months.

Our patient required prolonged follow-up of his hypogammaglobulinemia even after discharge from the hospital. He was receiving IVIG treatment weekly in combination with daily anakinra. A little over a year later, the patient is still working of his recovery from this near-fatal illness.

## Conclusions

HLH is a severe immune disorder characterized by dysregulation of the natural killer T-cell function, causing macrophage and lymphocyte activation with a subsequent cytokine storm that leads to organ failure and death. HLH can be familial or can be triggered by stimuli, mostly infections, malignancies, rheumatological diseases, or post-vaccination. The presentation includes fairly nonspecific symptoms such as fever, cytopenias, or liver dysfunction. HLH should be suspected in patients with those symptoms, especially in the setting of cytopenia and markedly elevated ferritin levels. The H-score helps predict the probability of HLH. Evidence of hemophagocytosis in bone marrow biopsy increases the likelihood of the diagnosis, but its absence does not rule it out. If suspected, early initiation of HLH targeted therapy with immunosuppressants including steroids may prevent irreversible organ damage and subsequent death.
